# MicroRNA-155 enhances the activation of Wnt/β-catenin signaling in colorectal carcinoma by suppressing HMG-box transcription factor 1

**DOI:** 10.3892/mmr.2022.12597

**Published:** 2022-01-12

**Authors:** Ying-Chun Wan, Tao Li, Yang-Dong Han, Hai-Yan Zhang, Hai Lin, Bin Zhang

Mol Med Rep 13: 2221-2228, 2016; DOI: 10.3892/mmr.2016.4788

Following the publication of this paper, it was drawn to the Editors’ attention by a concerned reader that certain of the western blot assay data shown in Figs. 1C and 6A were strikingly similar to data appearing in different form in another article written by different authors. Owing to the fact that the contentious data in the above article were already under consideration for publication prior to its submission to *Molecular Medicine Reports*, the Editor has decided that this paper should be retracted from the Journal. The authors were asked for an explanation to account for these concerns, but the Editorial Office did not receive a satisfactory reply. The Editor apologizes to the readership for any inconvenience caused.

